# Efficacy and safety of mirikizumab (LY3074828) in chronic plaque psoriasis: a systematic review and meta-analysis of randomized controlled trials

**DOI:** 10.3389/fmed.2025.1591787

**Published:** 2025-07-22

**Authors:** Taimoor Ashraf, Anand Kumar Malani, Dileep Kumar, Raja Subhash Sagar, Sahil Raj, Payal Kumari, Aanchal Kumari, Simran Bajaj, Muhammad Abdul Basit, Muhammad Murad, Vikash Kumar, Ayush Kumar

**Affiliations:** ^1^Department of Medicine, Nishtar Medical University, Multan, Pakistan; ^2^Department of Medicine, Liaquat University of Medical and Health Sciences, Jamshoro, Pakistan; ^3^Department of Medicine, Bahria University Medical and Dental College, Karachi, Pakistan; ^4^Department of Medicine, Jinnah Sindh Medical University, Karachi, Pakistan; ^5^Department of Medicine, Shaheed Mohtarma Benazir Bhutto Medical University, Larkana, Pakistan; ^6^Department of Medicine, Dow Medical College, Karachi, Pakistan; ^7^Department of Medicine, Vayodha Hospitals, Kathmandu, Nepal

**Keywords:** psoriasis, plaque psoriasis, mirikizumab, interleukin-23, psoriasis area and severity index

## Abstract

**Background:**

Chronic plaque psoriasis is a persistent inflammatory skin condition characterized by erythematous, scaly plaques, significantly impairing the quality of life of affected individuals. Mirikizumab, a humanized monoclonal antibody targeting interleukin-23 (IL-23) p19 subunit, has shown promising efficacy in managing moderate-to-severe cases of this disease. This meta-analysis aims to evaluate the efficacy and safety of mirikizumab in comparison to placebo or other active treatments in this patient population.

**Methods:**

A systematic review of randomized controlled trials (RCTs) was conducted. Eligible studies were identified through searches of major databases. The primary endpoint was clinical response measured by Psoriasis Area and Severity Index (PASI) improvement. Safety outcomes were evaluated based on the frequency of events. Data were pooled using a random-effects model to calculate relative risk and mean differences across studies.

**Results:**

Mirikizumab significantly increased the rates of achieving PASI 100, PASI 90, and PASI 75 compared to placebo. Mirikizumab also demonstrated superior efficacy in various secondary outcomes compared to placebo. Safety analysis indicated no significant differences in overall treatment-emergent adverse events (TEAEs) or serious adverse events (SAEs), although upper respiratory tract infections occurred more frequently in the mirikizumab group.

**Conclusion:**

Mirikizumab demonstrated a significant improvement in PASI scores compared to placebo and other treatments for chronic plaque psoriasis. Its IL-23 inhibition mechanism suggests a promising therapeutic option with a favorable safety profile. Further research could solidify its position in long-term psoriasis management.

**Systematic review registration:**

https://www.crd.york.ac.uk/PROSPERO/, identifier [CRD42024594843].

## Introduction

Psoriasis is a chronic, immune-mediated inflammatory skin disorder, with psoriasis vulgaris (plaque psoriasis) being its most common form. Characterized by well-defined, erythematous, and pruritic plaques with silvery scales, this condition is marked by excessive keratinocyte proliferation and abnormal differentiation (1–4). The hallmark of psoriasis is persistent inflammation, leading to epidermal hyperplasia (acanthosis) accompanied by immune cell infiltration, including dermal dendritic cells, T cells (notably the Th17 subset), neutrophils, and macrophages, along with marked neovascularization (5). Pathogenesis is primarily driven by pro-inflammatory cytokines such as IL-12, IL-17, and IL-23, which have led to the development of biological therapies targeting these cytokines and related inflammatory pathways for the treatment of plaque psoriasis (6). Although, rarely life-threatening, psoriasis affects over 125 million people globally (7). This non-communicable disease, along with other dermatological conditions, significantly impacts patients’ quality of life, leading to considerable financial, physical, social, and psychological burdens (8–14).

The identification of IL-17 and IL-23 as pivotal cytokines in the pathogenesis of psoriasis has revolutionized treatment approaches over the past decade. Therapies targeting these pathways have achieved unprecedented efficacy levels, surpassing earlier biologic therapies. Ustekinumab, one of the first biologics in this class, inhibits the shared p40 subunit of IL-12 and IL-23. However, emerging evidence, including gene expression studies in psoriatic lesions and animal models, highlights IL-23 as the primary driver of psoriasis, rather than IL-12. Consequently, newer biologics that selectively target the IL-23 p19 subunit have demonstrated superior PASI response rates and favorable safety profiles (15). Biological therapies, such as mirikizumab, are at the forefront of these new treatments.

Mirikizumab is a monoclonal antibody that targets the IL-23 cytokine by blocking its p19 subunit, thereby reducing inflammation and keratinocyte overgrowth (16). Clinical trials have shown that patients treated with mirikizumab experience significant improvement in their symptoms with minimal adverse effects (15, 17, 18). This meta-analysis aims to pool data from multiple randomized controlled trials to provide a comprehensive assessment of mirikizumab’s efficacy and safety, offering a clearer picture of its potential in revolutionizing psoriasis management. By focusing on immune modulation, these therapies represent a promising future for long-term disease control.

## Methods

### Search methodology and data collection

A thorough and systematic search of randomized controlled trials (RCTs) was performed using databases such as PubMed, Google Scholar, ClinicalTrials.gov, and the Cochrane Library up to October 2024. This analysis was carefully structured in accordance with the 2020 PRISMA recommendations, and the methodological quality was assessed using the AMSTAR tool (19, 20). Moreover, it was formally recorded in the PROSPERO registry under the ID CRD42024594843 (21). The search incorporated both Medical Subject Headings (MeSH) and relevant keywords, including “mirikizumab,” “LY3074828,” “IL-23 p19 inhibitor,” and terms related to psoriasis, such as “plaque psoriasis,” “chronic plaque psoriasis,” or “psoriasis vulgaris.” The selection process involved reviewing titles, abstracts, full-texts, and reference lists of the identified studies. Additionally, references from significant papers and review articles were scrutinized to find other relevant studies, without restrictions on language, ethnicity, or region. Following a systematic search, identified articles were reviewed, and duplicate entries were eliminated using EndNote Reference Manager. Two independent reviewers screened the titles and abstracts of studies meeting the eligibility criteria. Full texts of the shortlisted publications were then meticulously assessed. Data from the selected trials were extracted and systematically compiled into a structured data table. Key information included the lead author’s name, year of publication, NCT number, total sample size, participant demographics (age and sex), baseline characteristics, dosage and frequency of mirikizumab administration, delivery method, and primary outcomes. Discrepancies in data extraction were resolved by discussion or, when necessary, by consulting a third reviewer.

### Study selection criteria and measured outcomes

#### Inclusion criteria

The research adhered to strict eligibility criteria, which included the following:

(a) Adults aged 18–75 years with a confirmed diagnosis of chronic plaque psoriasis for at least 6 months prior to enrollment. Eligible participants also had to exhibit psoriasis affecting 10% or more of their body surface area (BSA), a Psoriasis Area and Severity Index (PASI) score of 12 or higher, and a static Physician’s Global Assessment (sPGA) score of at least 3 at both screening and baseline.
(b) Multicenter randomized controlled trials (RCTs) with a 16-week induction phase, including at least one intervention group treated with mirikizumab and a placebo comparison group.
(c) Studies reporting outcomes relevant to the research questions, with no restrictions on dosage.

#### Exclusion criteria

Studies were excluded based on stringent criteria to ensure the validity and relevance of the evidence. Non-randomized or non-controlled trials, including observational studies, case reports, and uncontrolled trials, were deemed ineligible. Additionally, studies lacking critical outcome data—such as PASI 100, PASI 90, PASI 75, sPGA improvements, or patient-reported outcomes—or those focusing solely on pharmacokinetic or pharmacodynamic evaluations without efficacy or safety endpoints, were excluded. Unpublished studies, abstracts without full-text availability, and studies with inadequate methodological details—such as unclear randomization processes, absence of a control group, undefined eligibility criteria, or incomplete reporting of outcomes—were also excluded, as such deficiencies precluded a robust risk-of-bias assessment.

### Outcomes of interest

The main objective of this meta-analysis was to assess the efficacy of mirikizumab in improving skin outcomes compared to placebo at week 16, with the primary endpoints being the proportion of patients achieving PASI 100 (complete skin clearance), PASI 90 (90% clearance), and PASI 75 (75% clearance). Additional outcomes included sPGA 0 (clear) or 1 (almost clear) with a ≥2-point improvement from baseline, reduction in the Palmoplantar Psoriasis Severity Index (PPASI) for those with palmoplantar involvement, and improvement in the Nail Psoriasis Severity Index (NAPSI) for those with nail psoriasis. The analysis also evaluated the percentage of patients with ≤1% BSA affected by psoriasis. Patient-reported outcomes and health-related quality of life were considered, focusing on the number of participants achieving a Psoriasis Symptoms and Signs (PSS) score of 0 (no symptoms) and those with a Dermatology Life Quality Index (DLQI) score of 0 or 1, along with a ≥5-point improvement in individuals with an initial DLQI of ≥5. Safety monitoring was thorough, focusing on treatment-emergent adverse events (TEAEs), serious adverse events (SAEs), and particular adverse events of interest.

### Statistical analysis

All statistical analyses were performed using Review Manager 5.3 (RevMan 5.3) software. Dichotomous outcomes were analyzed using the Mantel–Haenszel method, chosen for its reliability in combining data from multiple studies with binary results, with results expressed as risk ratios (RRs) and 95% confidence intervals (CIs) to measure the relative likelihood of an event occurring in one group compared to another. Continuous variables were analyzed using the inverse variance method to calculate mean differences (MDs) with 95% CIs, a method selected for its precision in weighting studies based on the inverse of their variance, ensuring that larger studies with more precise estimates contributed proportionally more to the analysis. To account for potential variations across studies, such as differences in populations, methodologies, or interventions, a random-effects model was applied, offering more generalized and robust estimates by assuming that the true effect size may vary between studies. Heterogeneity was assessed using Cochrane’s *Q* statistic and Higgins *I*^2^ statistic, with the *Q* statistic detecting the presence of heterogeneity (*p* < 0.10 indicating significance) and the *I*^2^ statistic quantifying the proportion of total variation due to heterogeneity rather than chance, interpreted as low (<50%), moderate (50–75%), or substantial (>75%) heterogeneity. These methods ensured a comprehensive understanding of variability in the pooled results and justified the application of the random-effects model (22). When significant heterogeneity was identified, sensitivity analyses were performed by systematically excluding individual studies one at a time to assess their influence on the overall results and to pinpoint potential sources of variability, ensuring a more thorough and reliable interpretation of the findings. Statistical significance was determined by a *p* < 0.05.

### Assessment of risk of bias

The risk of bias for each included study was assessed using the RoB tool recommended by the Cochrane collaboration. This tool evaluates several domains, including random sequence generation, allocation concealment, blinding of participants and personnel, blinding of outcome assessment, incomplete outcome data, selective reporting, and other biases. Each domain was rated as low, high, or unclear risk based on the information provided in the study protocols and reports. The assessment aimed to ensure the methodological quality and reliability of the evidence derived from the included randomized controlled trials (23).

## Results

### Study screening and selection

**Figure 1:**
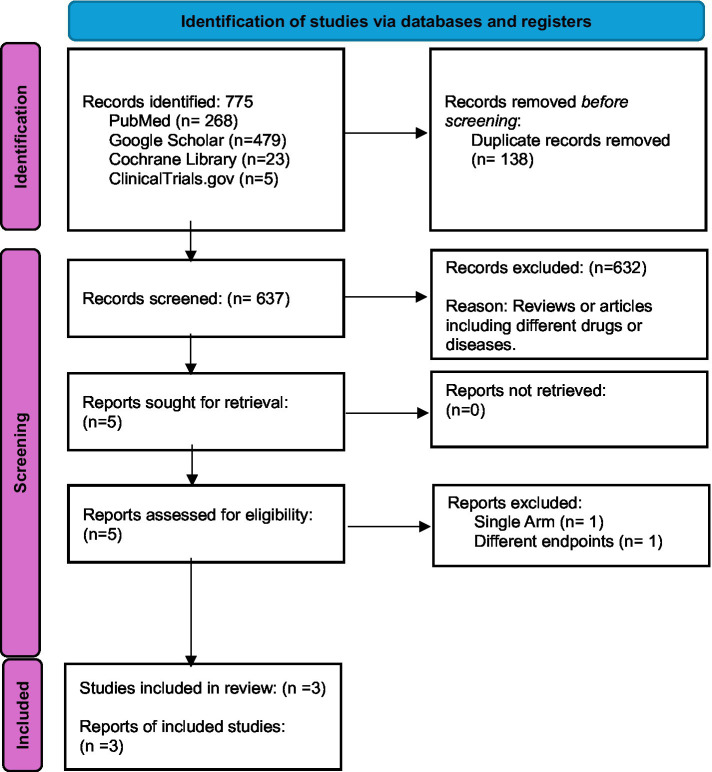
PRISMA flow diagram.

Initially, 775 records were identified from various databases and registers, including PubMed (268), Google Scholar (479), Cochrane Library (23), and ClinicalTrials.gov (5). After removing 138 duplicates, 637 records were screened. Of these, 632 were excluded, primarily due to being reviews or involving unrelated drugs or diseases. Five reports were sought for retrieval, and all were successfully retrieved. Following further assessment, two reports were excluded resulting in three studies being included in the final review (15, 17, 18) (Figure 1).

### Baseline and study characteristics

The baseline and study characteristics of the included randomized controlled trials (RCTs) are described in detail. Altogether, 1,752 participants took part in the three trials, with 1,481 receiving mirikizumab and 271 placed in the placebo group. Participants’ average age ranged from 43.8 to 49.2 years. The majority were male, accounting for 69.3% of the total population (1,214 men), while 538 were female (30.7%). These multicenter trials consistently administered mirikizumab or placebo subcutaneously, with dosing schedules of either once every 8 weeks (Q8W) or every 4 weeks (Q4W), and dosages varying between 30 mg and 300 mg (Table 1).

**Table 1:** Study and baseline characteristics.

Study, year, and NCT	Phase	Groups	No. of participants (*n*)	Sex (M/F)	Age (years) (mean ± SD)	Psoriasis duration (mean ± SD)	PASI (mean ± SD)	% BSA involvement (mean ± SD)	Dosing	Follow up
Reich, K. et al., 2019 (15), NCT02899988	Phase II	Mirikizumab	51	39/12	46 ± 13.3	20.4 ± 13.5	21.0 ± 8.4	27.3 ± 15.8	SC (Q8W)	16 weeks
		(30 mg)								
		(100 mg)	51	35/16	49.2 ± 13.2	18.6 ± 11.3	20.3 ± 8.0	26.5 ± 16.5	SC (Q8W)	
		(300 mg)	51	36/15	47.5 ± 13.2	18.1 ± 12.7	18.4 ± 6.9	21.3 ± 10.3	SC (Q8W)	
		Placebo	52	42/10	46 ± 12.4	18.0 ± 9.8	19.7 ± 7.4	26.4 ± 17.5	SC (Q8W)	
Blauvelt, A. et al., 2022 (17), NCT03482011	Phase III	Mirikizumab	423	299/124	46.4 ± 13.6	17.7 ± 11.5	23.5 ± 10.1	31.9 ± 19.4	SC (Q4W)	16 weeks
		(250 mg)								
		Placebo	107	74/33	45.7 ± 13.7	17.0 ± 10.9	22.3 ± 9.8	31.3 ± 20.3	SC (Q4W)	
Papp, K. et al., 2023 (18), NCT03535194	Phase III	Mirikizumab	451	311/140	47 ± 14	17·4 ± 12·0	20.9 ± 7.9	27.2 ± 15.3	SC (Q4W)	16 weeks
		(250 mg)								
		(250 mg)	454	296/158	45.8 ± 13	17·2 ± 12·5	21.1 ± 8.3	28.1 ± 17.0	SC (Q4W)	

### Assessment of quality

**Figure 2:**
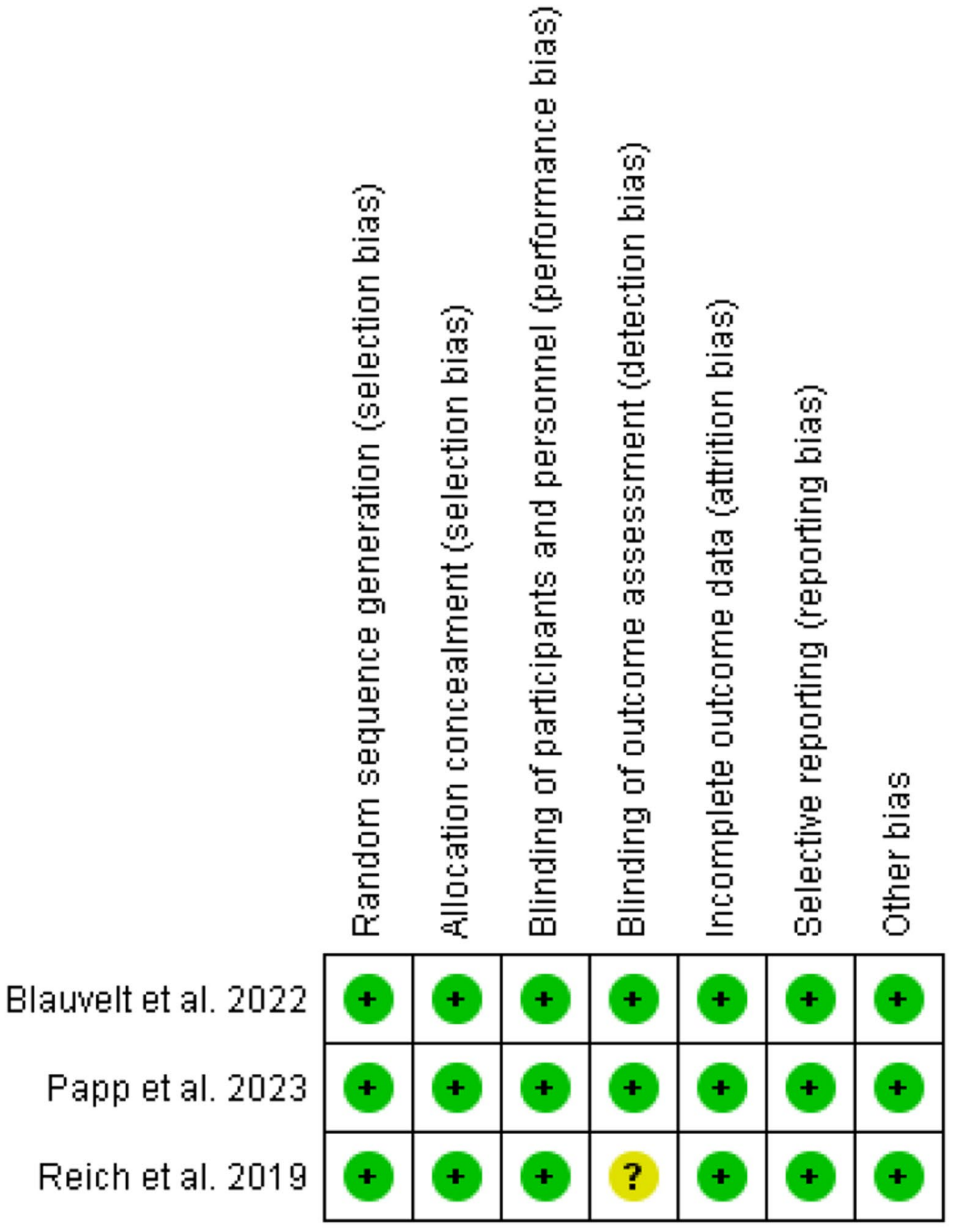
Risk of bias summary.

**Figure 3:**
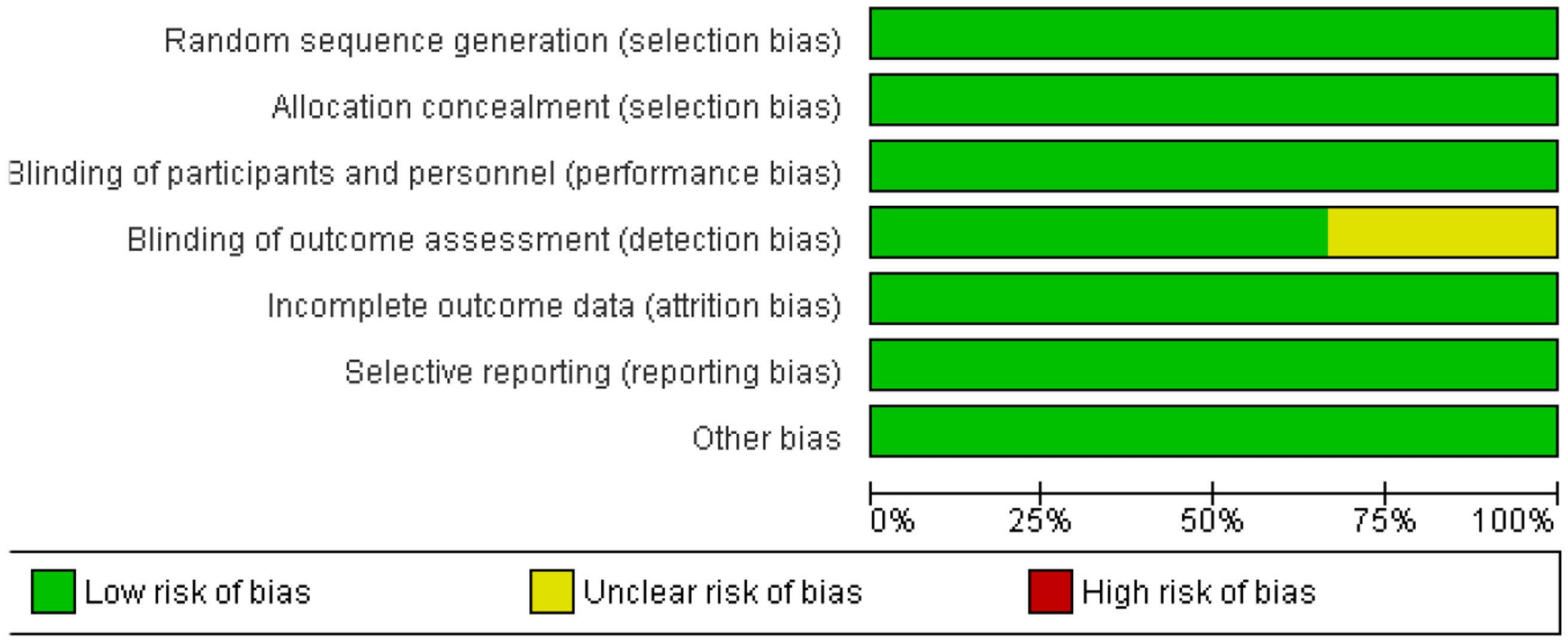
Risk of bias graph.

The risk of bias across the included studies was generally low. Most trials demonstrated a low risk of bias in key domains, including random sequence generation, allocation concealment, blinding of participants, personnel, and outcome assessors. However, one study showed an unclear risk of bias in the blinding of outcome assessment. No significant issues were found in terms of incomplete outcome data, selective reporting, or other biases. Overall, the studies were of high methodological quality, ensuring the reliability of the findings. Figures 2, 3 depict a complete assessment.

### Primary outcomes

#### PASI

**Figure 4:**
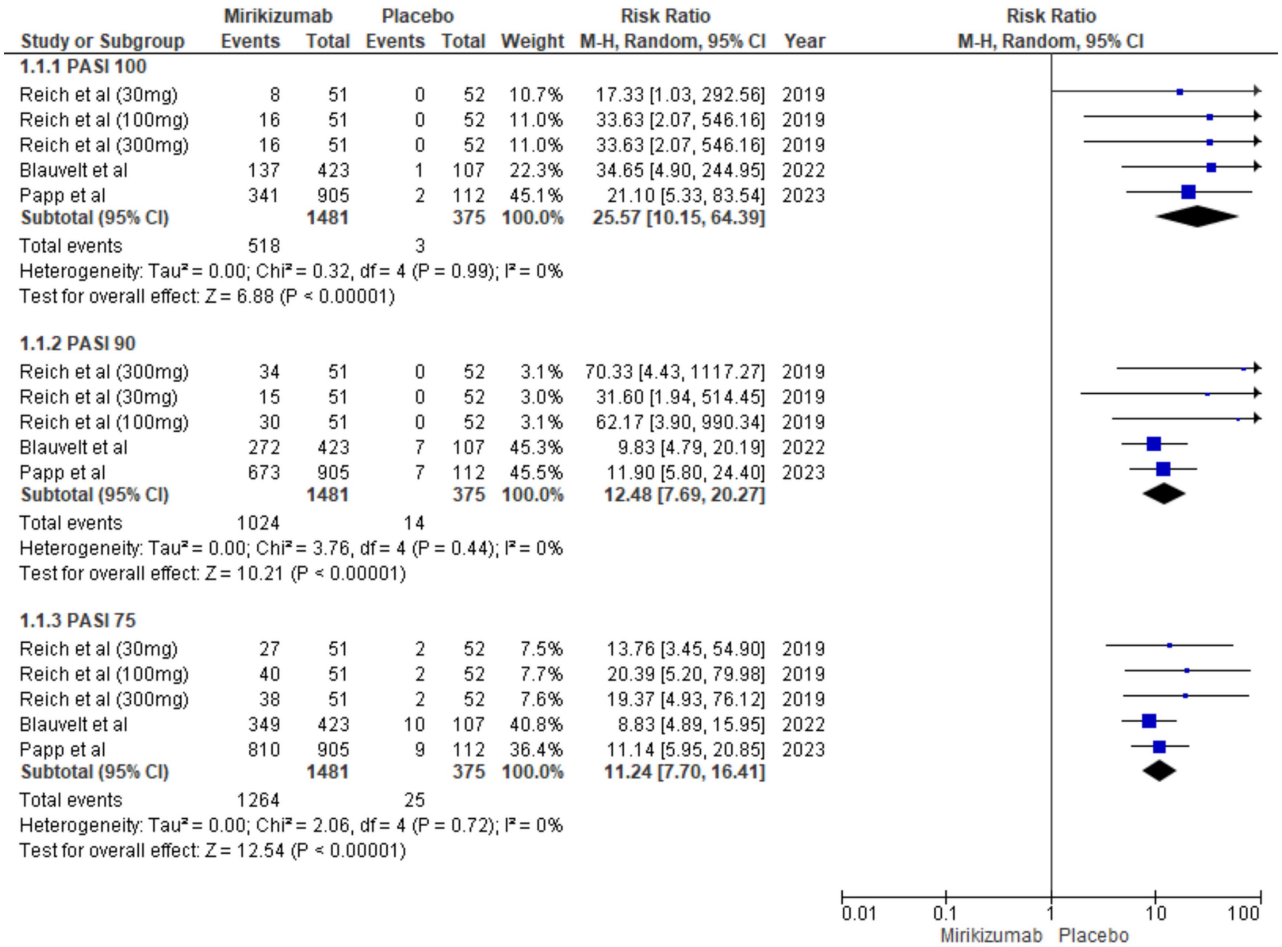
Forest plot for PASI.

The proportion of patients achieving PASI 75, PASI 90, and PASI 100 was significantly higher in the mirikizumab group compared to placebo, with risk ratios of 11.70 (95% CI: 7.70–16.41, *p* < 0.00001), 12.48 (95% CI: 7.69–20.27, *p* < 0.00001), and 25.57 (95% CI: 10.15–64.39, *p* < 0.00001), respectively. No heterogeneity was observed across these outcomes (*I*^2^ = 0%) (Figure 4).

### Secondary outcomes

#### BSA involvement <1%

**Figure 5:**
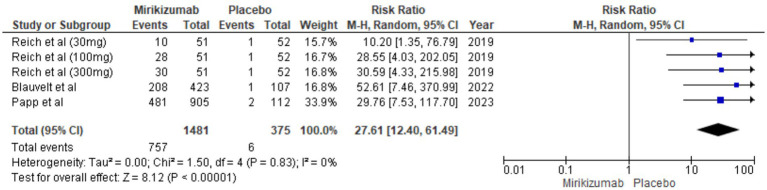
Forest plot for <1% involvement of BSA.

A significantly higher proportion of patients on mirikizumab achieved <1% BSA involvement compared to placebo (RR 27.61, 95% CI: 12.40–61.49, *p* < 0.00001), with no heterogeneity observed (*I*^2^ = 0%) (Figure 5).

### sPGA score

**Figure 6:**
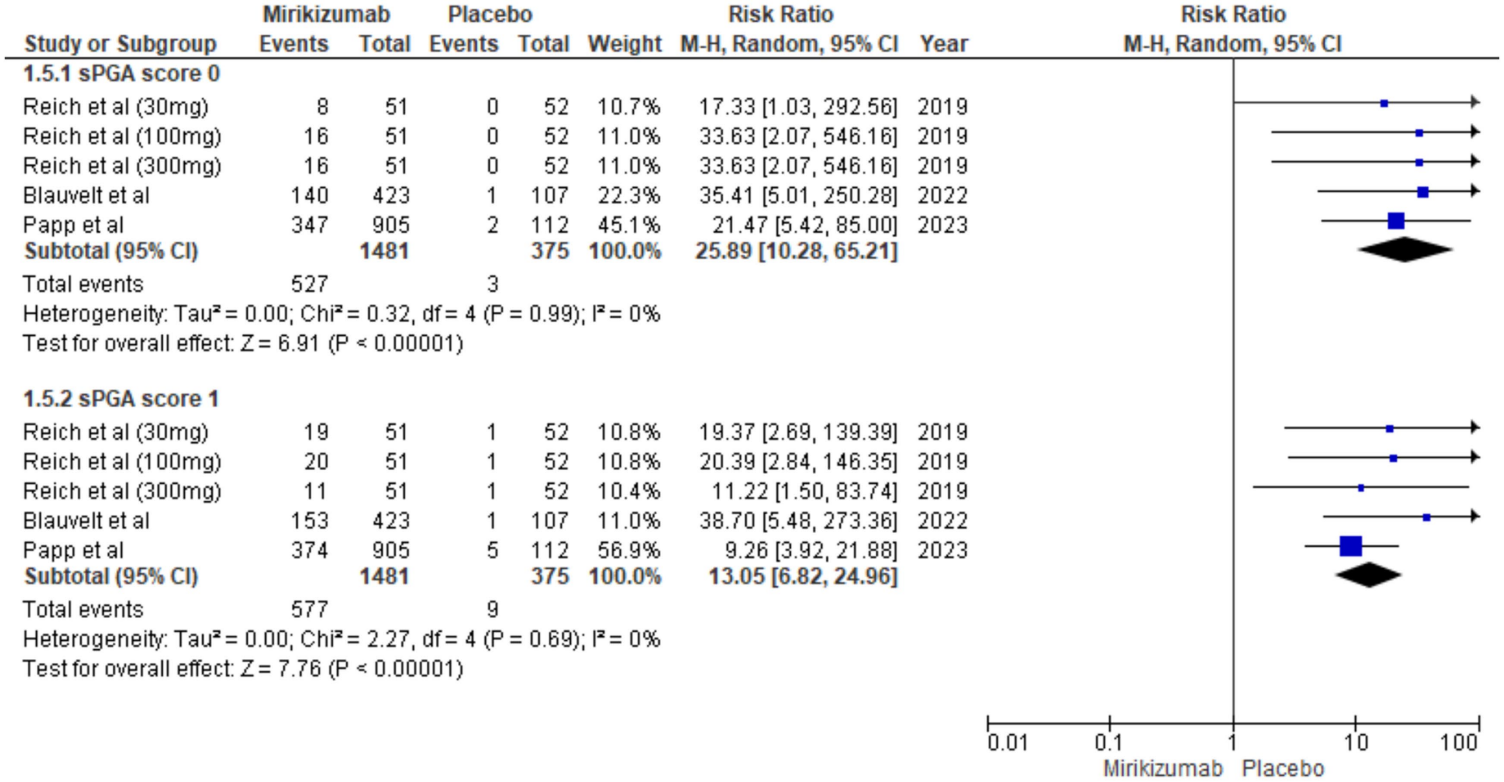
Forest plot for sPGA score.

Mirikizumab was significantly more effective than placebo in achieving both sPGA scores of 0 (RR 25.89, 95% CI: 10.28–65.21, *p* < 0.00001) and 1 (RR 13.05, 95% CI: 6.82–24.96, *p* < 0.00001), with no heterogeneity observed for either outcome (*I*^2^ = 0%) (Figure 6).

#### PPASI

**Figure 7:**
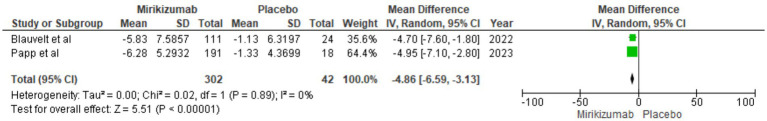
Forest plot for PPASI.

The analysis indicated a significant reduction in PPASI scores from baseline for mirikizumab participants, with an MD of −4.86 (95% CI: −6.59 to −3.13, *p* < 0.00001). Heterogeneity was absent (*I*^2^ = 0%) (Figure 7).

#### NAPSI

**Figure 8:**
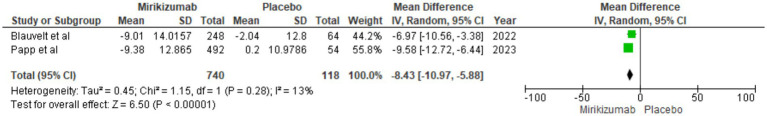
Forest plot for NAPSI.

Mirikizumab treatment resulted in a notable decrease in NAPSI scores, showing an MD of −8.43 (95% CI: −10.97 to −5.88, *p* < 0.00001). This improvement occurred with minimal heterogeneity (*I*^2^ = 13%) (Figure 8).

#### DLQI score of 0 or 1

**Figure 9:**
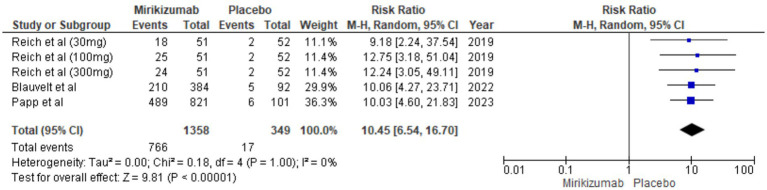
Forest plot for DLQI score of 0 or 1.

There was a significant difference in DLQI outcomes, with mirikizumab participants showing considerable improvement compared to the placebo group. The RR for this outcome was 10.45 (95% CI: 6.54–16.70, *p* < 0.00001), and no heterogeneity was detected (*I*^2^ = 0%) (Figure 9).

#### PSS score of 0

**Figure 10:**
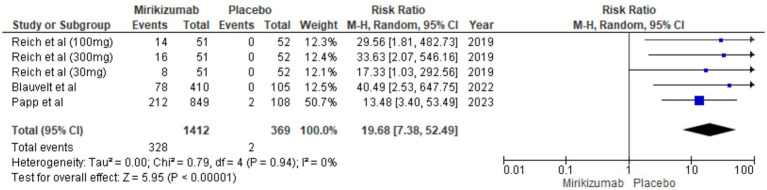
Forest plot for PSS score of 0.

The findings demonstrated a significant difference in PSS improvements, with mirikizumab recipients showing substantial enhancement compared to those on placebo. The RR was 19.68 (95% CI: 7.38–52.49, *p* < 0.00001), and the absence of heterogeneity (*I*^2^ = 0%) reinforces the consistency of these results (Figure 10).

#### Overall TEAES

**Figure 11:**
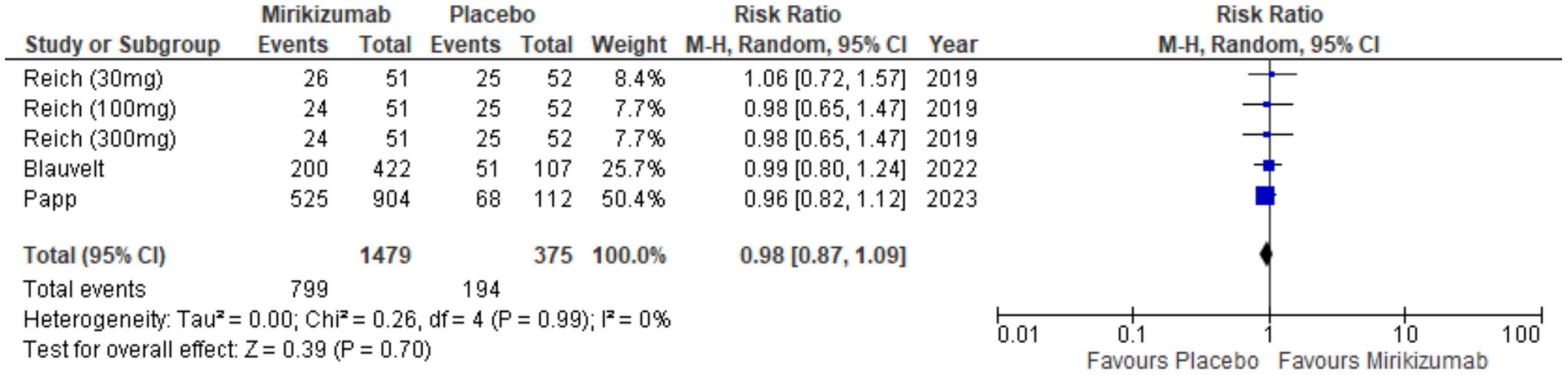
Forest plot for overall TEAEs.

The analysis showed no significant link between mirikizumab and TEAEs, with 799 incidents in 1,479 patients on mirikizumab versus 194 in 375 patients on placebo. The RR was 0.98 (95% CI: 0.87–1.09) and the *p*-value was 0.70, indicating no meaningful difference between groups. Heterogeneity was also absent (*I*^2^ = 0%) (Figure 11).

#### SAEs

**Figure 12:**
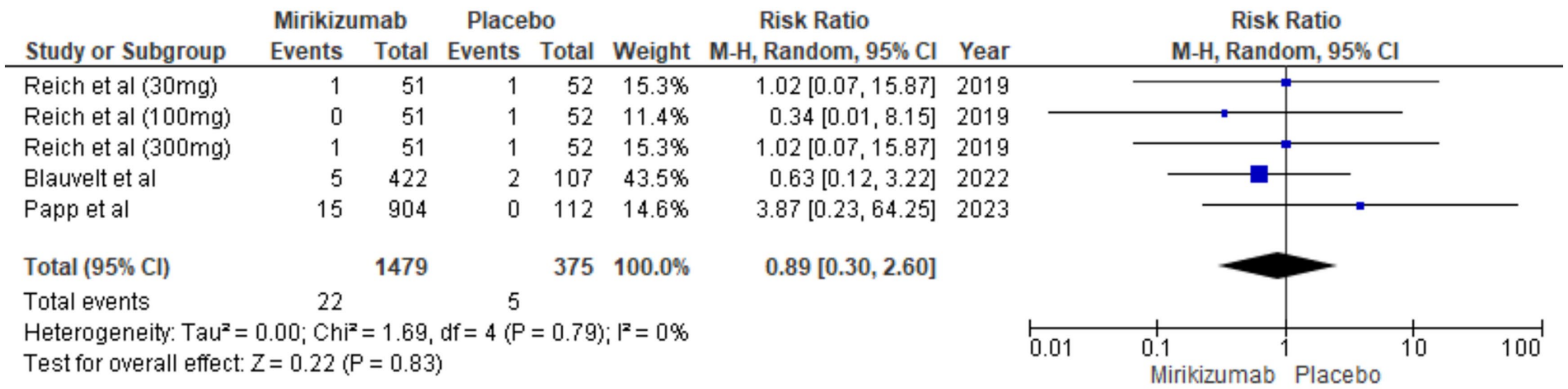
Forest plot for SAEs.

There were 22 SAEs in the mirikizumab group compared to 5 in the placebo group. The RR was 0.89 (95% CI: 0.30–2.60) with a *p*-value of 0.83, showing no statistically significant difference. Additionally, there was no heterogeneity among studies (*I*^2^ = 0%) (Figure 12).

### Adverse events of concern

Adverse events of concern showed no significant differences between the mirikizumab and placebo groups, with the following *p* values: arthralgia (*p* = 0.98), back pain (*p* = 0.11), diarrhea (*p* = 0.53), headache (*p* = 0.82), hypertension (*p* = 0.31), total infections (*p* = 0.29), injection-site pain (*p* = 0.80), mortality (*p* = 0.55), nasopharyngitis (*p* = 0.87), neoplasms (*p* = 0.83), pruritus (*p* = 0.26), and upper respiratory infection (*p* = 0.07). Heterogeneity was low for all outcomes (Supplementary Figures 1–12).

## Discussion

This study represents the first comprehensive meta-analysis evaluating the efficacy of mirikizumab for chronic plaque psoriasis. The findings demonstrate that mirikizumab significantly outperformed placebo across various efficacy measures, reflecting its robust ability to improve clinical and quality-of-life outcomes in patients with chronic plaque psoriasis. These results underscore its therapeutic potential as an effective treatment option. In terms of safety, there was no significant difference in the overall incidence of TEAEs or SAEs between mirikizumab and placebo groups. Importantly, no heterogeneity was detected across these outcomes, further strengthening the consistency of the results. These results consistently highlight the superior effectiveness of mirikizumab compared to placebo in clearing psoriatic lesions, reaffirming its potential as a highly efficacious therapeutic option.

Over the past decade, there has been a notable gain in our comprehension of the function played by cytokines associated with T-helper cell 17 and IL-23 in the pathogenesis of psoriasis. Therefore, compared to conventional biological treatments, therapies that target interleukin-17 and interleukin-23 have shown superior efficacy in a broader patient population (24, 25). Various cell kinds, in particular macrophages, plasmacytoid dendritic cells, keratinocytes, and natural killer T cells, are responsible for producing IL-23, a pivotal player in the pathophysiology of psoriasis. IL-23 drives the maturation of T-cells into Type 17 T-helper cells, further fueling the inflammatory process. Subsequently, IL-17A and IL-17F from Th17 cells activate keratinocytes, prompting their proliferation and secretion of pro-inflammatory cytokines, chemokines, and antimicrobial peptides. This initiates a feedback loop, attracting more innate and adaptive immune cells to intensify inflammation. Moreover, this cascade stimulates angiogenic mediators and prompts the release of endothelial adhesion molecules, facilitating the migration of immune cells into psoriatic lesions (7, 26).

Clinical trials have demonstrated auspicious results for mirikizumab by selectively targeting the IL-23 binding site, thereby inhibiting Th17 cells and subsequently diminishing the levels of IL-17A and IL-17F. This mechanism significantly mitigates inflammation and improves the appearance of psoriatic skin lesions (15, 17, 18). A RCT aimed to assess the clinical effectiveness, tolerability, and safety of mirikizumab for treating ulcerative colitis reported a decrease in circulating IL-17 levels. This observation corresponds with the anticipated consequences of inhibiting the IL-23 pathway (27). The substantial clinical response rates achieved with mirikizumab are congruent with findings from investigations into other biologics targeting IL-23, which have shown superior efficacy when contrasted with TNF, IL-17, and IL-12/IL-23 inhibitors (18). Furthermore, IL-23 inhibitors, such as mirikizumab, have a major therapeutic benefit over other biologic treatments since they target upstream cytokines rather than downstream ones like TNF-α and IL-17, necessitating less frequent dosage (28, 29).

Mirikizumab, by selectively targeting the IL-23 binding site, has demonstrated remarkable efficacy in treating chronic plaque psoriasis. Clinical trials and this meta-analysis confirm its superiority over placebo across multiple outcomes, including achieving PASI 100, PASI 90, and PASI 75, with significant response rates of 35.0, 69.1, and 85.3%, respectively. Additionally, mirikizumab was highly effective in reducing BSA involvement to <1% and achieving sPGA scores of 0 and 1, with no heterogeneity observed across studies. Secondary outcomes, such as reductions in PPASI, NAPSI, and improvements in DLQI and PSS scores, further underscore its therapeutic benefits. These findings align with the well-established role of IL-23 in the pathogenesis of psoriasis and highlight mirikizumab’s ability to disrupt the inflammatory cascade mediated by IL-23 and Th17 cytokines (18). Given its robust efficacy, favorable safety profile, and infrequent dosing requirements, mirikizumab emerges as a promising alternative treatment option for managing chronic plaque psoriasis. The tolerability and safety profiles between the mirikizumab cohort and the placebo cohort revealed no significant discrepancies in our study, underscoring the commendable safety record of mirikizumab in the treatment of psoriasis. Although the incidence of upper respiratory tract infections was slightly higher in the mirikizumab cohort, with a *p*-value of 0.07, the difference was not statistically significant. Notably, RCT investigating mirikizumab consistently reported that most infections, including upper respiratory tract infections, were mild to moderate in severity (18).

While mirikizumab initially demonstrated significant efficacy in psoriasis, with strong results in skin clearance and patient-reported outcomes, its development has since shifted toward IBD, including ulcerative colitis and Crohn’s disease. By selectively inhibiting the IL-23/Th17 axis, mirikizumab reduces key inflammatory mediators like IL-17A and IL-17F, which play a central role in IBD pathogenesis (30). Clinical trials have confirmed its ability to improve endoscopic healing, clinical response rates, and long-term remission, with a safety profile comparable to placebo—findings supported by multiple RCTs showing no significant differences in adverse events between mirikizumab and placebo groups (27, 31, 32). This upstream targeting of IL-23 may offer advantages over conventional biologics, including sustained efficacy and less frequent dosing. However, despite its proven benefits in plaque psoriasis, mirikizumab is not yet being pursued for dermatological use, reflecting the evolving IL-23 inhibitor landscape. With established agents like guselkumab and risankizumab already dominating psoriasis treatment, mirikizumab’s strategic focus on IBD addresses critical unmet needs in gastroenterology, positioning it as a promising therapeutic option in this space (33).

The present meta-analysis has several limitations. First, the analysis included only three RCTs, which may limit the generalizability of the findings. These studies consisted of one phase II trial investigating three doses (30 mg, 100 mg, and 300 mg) and two phase III studies that utilized a 250 mg dose. The variation in dosing regimens could potentially influence the results. Additionally, the follow-up period was restricted to the 16-week induction phase, leaving the long-term safety and efficacy of mirikizumab unaddressed. Given that psoriasis is a chronic, relapsing disease requiring sustained management, long-term data are essential to evaluate whether the observed benefits of mirikizumab are durable over time and to identify any delayed adverse events. Future trials should incorporate extended follow-up periods beyond the induction phase to better assess the sustainability of clinical response, durability of remission, long-term safety, and optimal maintenance dosing strategies. These data are crucial for informing real-world clinical decisions and aligning treatment approaches with the chronic nature of psoriasis. Moreover, the included studies employed different dosing schedules (Q4W and Q8W), which have introduced variability in treatment response, complicating direct comparisons and interpretation of pooled outcomes. Furthermore, this study compared mirikizumab only to placebo and did not include head-to-head comparisons with other biologic agents such as IL-23 p19 inhibitors (e.g., guselkumab, risankizumab), IL-17 inhibitors, or IL-12/23 inhibitors, limiting the ability to contextualize its efficacy and safety within the broader therapeutic landscape. While direct comparative trials are ideal, the feasibility of network meta-analyses should be explored in future research to indirectly compare mirikizumab with other established biologics. Despite extensive efforts to minimize publication bias, it cannot be entirely ruled out, particularly since all included studies were industry-sponsored. Consequently, larger studies with extended follow-up periods are necessary to confirm these findings and generalize them to broader populations. Additionally, further research on variable dosing frequencies is needed to develop personalized treatment strategies.

## Conclusion

This meta-analysis demonstrates that mirikizumab significantly improves psoriasis symptoms compared to placebo, achieving higher rates of PASI 100, PASI 90, and PASI 75, along with reductions in BSA involvement, PPASI, NAPSI, and improvements in DLQI and PSS scores. It also exhibits a favorable safety profile, with no significant differences in TEAEs or SAEs between treatment and control groups. The consistency of findings across outcomes, reflected by the lack of significant heterogeneity, further reinforces the robustness of the evidence. Despite certain limitations, mirikizumab emerges as a promising therapeutic option for moderate-to-severe plaque psoriasis. Clinicians may consider mirikizumab as an effective and well-tolerated IL-23 p19 inhibitor that can offer meaningful improvements in both clinical and patient-reported outcomes, particularly in patients who are candidates for biologic therapy and require rapid disease control.

## Data Availability

The original contributions presented in the study are included in the article/Supplementary material, further inquiries can be directed to the corresponding author.
