# Supplementary material for: Efficacy and safety of mirikizumab (LY3074828) in chronic plaque psoriasis: a systematic review and meta-analysis of randomized controlled trials
Source: Front Med (Lausanne). 2025 Jul 22;12:1591787. doi: 10.3389/fmed.2025.1591787 (PMC12321557; doi:10.3389/fmed.2025.1591787)

**Supplementary Material**

**Supplementary Figure 1**: Forest plot for Arthralgia

**Supplementary Figure 2:** Forest plot for Back Pain

**Supplementary Figure 3:** Forest plot for Diarrhoea

**Supplementary Figure 4:** Forest plot for Headache

**Supplementary Figure 5:** Forest plot for Hypertension

**Supplementary Figure 6:** Forest plot for Total Infections

**Supplementary Figure 7:** Forest plot for Injection-Site Pain

**Supplementary Figure 8:** Forest plot for Mortality

**Supplementary Figure 9:** Forest plot for Nasopharyngitis

**Supplementary Figure 10:** Forest plot for Neoplasms

**Supplementary Figure 11:** Forest plot for Pruritus

**Supplementary Figure 12:** Forest plot for Upper Respiratory Infection

**Supplementary Figure 1**: Forest plot for Arthralgia


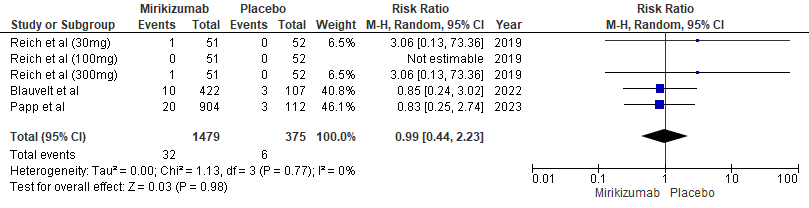


**Supplementary Figure 2:** Forest plot for Back Pain


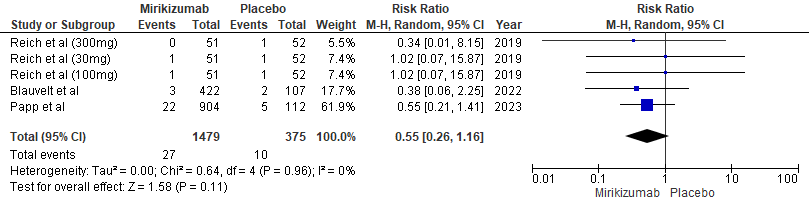


**Supplementary Figure 3:** Forest plot for Diarrhoea


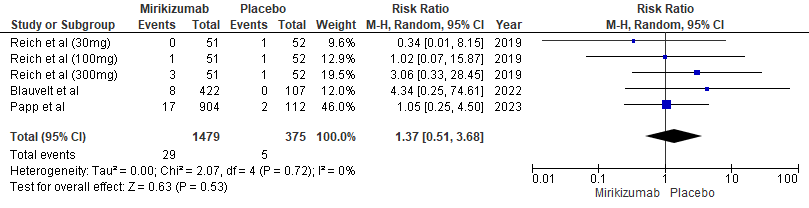
**Supplementary Figure 4:** Forest plot for Headache

**
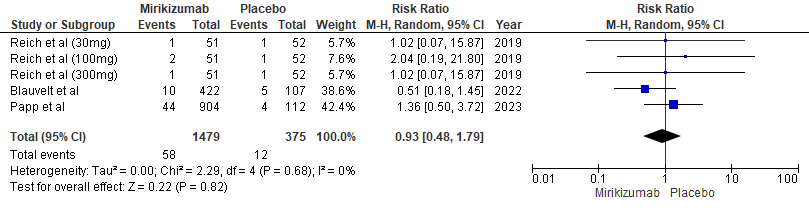
**

**Supplementary Figure 5:** Forest plot for Hypertension


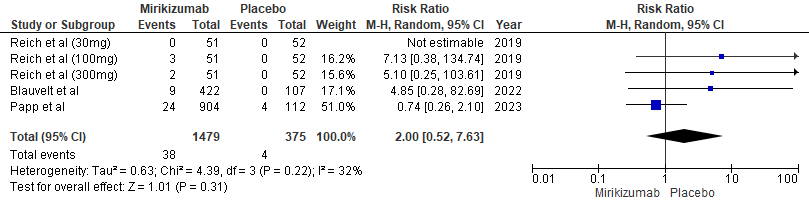


**Supplementary Figure 6:** Forest plot for Total Infections


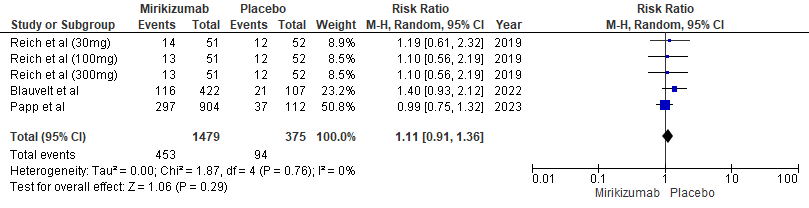


**Supplementary Figure 7:** Forest plot for Injection-Site Pain


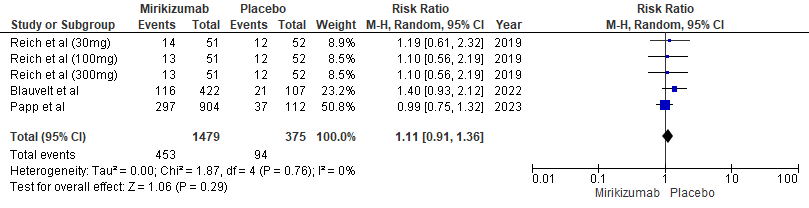


**Supplementary Figure 8:** Forest plot for Mortality


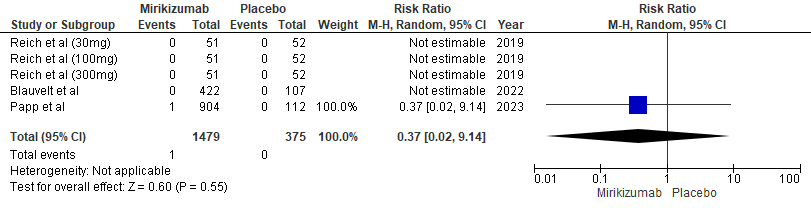


**Supplementary Figure 9:** Forest plot for Nasopharyngitis


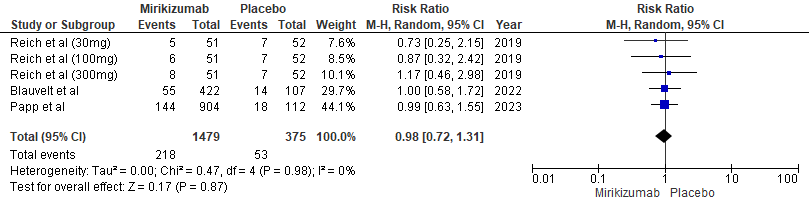


**Supplementary Figure 10:** Forest plot for Neoplasms


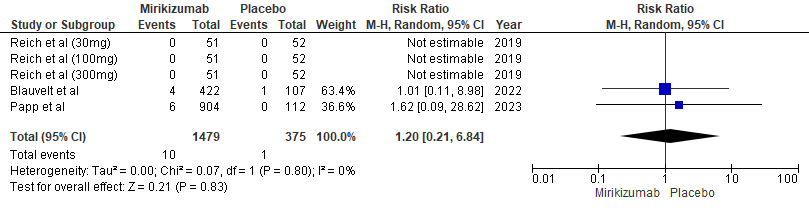


**Supplementary Figure 11:** Forest plot for Pruritus


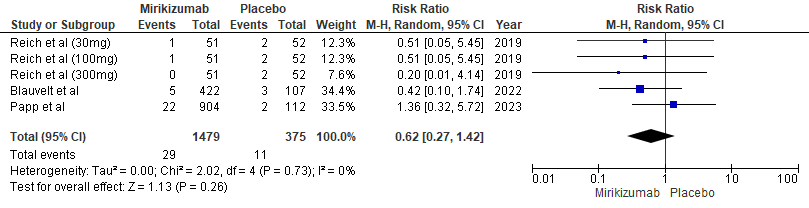


**Supplementary Figure 12:** Forest plot for Upper Respiratory Infection


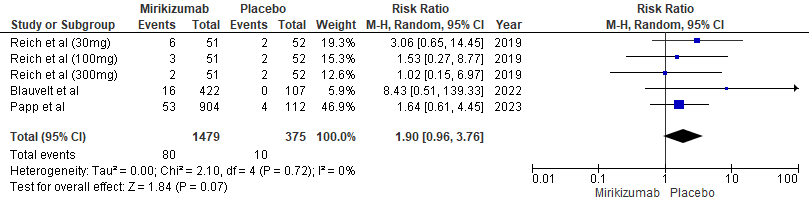

Supplement: Supplementary file 1 [file Data_Sheet_1.docx]
